# Developing and Integrating Regular Training in Serious Incident Investigations and Coroner’s Inquests Into the Higher Trainees Teaching at Kent and Medway NHS and Social Care Partnership Trust (KMPT)

**DOI:** 10.1192/bjo.2025.10291

**Published:** 2025-06-20

**Authors:** Maria Moisan, Chiara Rubino, Rachel Daly

**Affiliations:** Kent and Medway NHS and Social Care Partnership Trust, Maidstone, United Kingdom

## Abstract

**Aims:** This project aims to develop and integrate regular training on Serious Incident (SI) Investigations and Coroner’s Inquests into the Higher Trainees Teaching at Kent and Medway NHS and Social Care Partnership Trust (KMPT). The goal is to enhance trainees’ understanding and confidence in these critical areas, ultimately improving patient safety and supporting psychiatric trainees in their professional development.

**Methods:** Using Quality Improvement (QI) methodology, the project began with a baseline survey to assess trainees’ knowledge and confidence regarding SI investigations and Coroner’s Inquests. Based on identified needs, an Initial Training Event was held in November 2023, which included sessions on SI investigation processes, thematic reviews of suicides, patient safety, and involvement in investigations. The second QI cycle focused on developing and delivering a tailored training programme for Core and Higher Trainees in January 2024. This programme consisted of two sessions: “Introduction to Legal Services HM Coroner” and “Managing Serious Incidents”. Feedback from trainees was gathered through questionnaires to evaluate the effectiveness of the training.

**Results:** The baseline survey (April–May 2023) showed that 71.88% of respondents had limited understanding of SI investigations, with 87.5% expressing interest in further training. The Initial Training Event in November 2023 had 47 attendees, with 92.86% expressing a need for additional training. The tailored training programme in January 2024 had 20 attendees, with 100% of respondents indicating that the training would improve patient safety in their clinical practice. All trainees reported a better understanding of the Coroner’s Inquest process, and 100% agreed that the training should be repeated annually. Notably, the SI investigation process, including Root Cause Analysis (RCA), is now being replaced by the Patient Safety Incident Response Framework (PSIRF), which represents a shift toward a more flexible, learning-focused approach to managing patient safety incidents. The results from the baseline survey and the initial training event were published in BJPsych and presented at the International Congress RCPsych in June 2024.

**Conclusion:** The project successfully identified a significant gap in training regarding SI investigations and Coroner’s Inquests for psychiatric trainees at KMPT. The first two cycles of the QI process have demonstrated positive outcomes, and the need for regular, ongoing training has been clearly established. As a result, this training is now integrated into the Higher Trainees Teaching programme. Future considerations include evaluating feedback from the 2025 training session and potentially introducing Mock Coroner sessions and protocols for trainee involvement in SI investigations, under the new PSIRF framework.

